# Biofilm formation and dispersal of *Staphylococcus aureus* wound isolates in microtiter plate-based 2-D wound model

**DOI:** 10.1016/j.heliyon.2024.e33872

**Published:** 2024-07-02

**Authors:** José Manuel Outomuro Ruiz, Erik Gerner, Shadi Rahimi, Leonarda Achá Alarcón, Ivan Mijakovic

**Affiliations:** a Division of Systems and Synthetic Biology, Department of Life Sciences, Chalmers University of Technology, SE-41296, Gothenburg, Sweden; b Mölnlycke Health Care AB, SE-41502, Gothenburg, Sweden; c Gothenburg University, SE-40530, Gothenburg, Sweden; d The Novo Nordisk Foundation Center for Biosustainability, Technical University of Denmark, DK-2800, Kgs. Lyngby, Denmark

**Keywords:** Biofilm formation, Biofilm dispersal, *Staphylococcus aureus*, Static wound model

## Abstract

Biofilm-associated wound infections in diabetic and immunocompromised patients are an increasing threat due to rising antibiotic resistance. Various wound models have been used to screen for efficient antiinfection treatments. However, results from *in vitro* models do not always match *in vivo* results, and this represents a bottleneck for development of new infection treatments. In this study, a static 2-D microtiter plate-based biofilm model was tested for growing clinically relevant *Staphylococcus aureus* wound isolates in various operating conditions, seeking to identify an optimal setup that would yield physiologically relevant results. Specifically, the tested variables included wound-mimicking growth media, precoating of surface with different proteins, multiwell plates with various surface properties, and the effect of bacterial pre-attachment step. Our results indicated that protein precoating is a key factor for supporting biofilm growth. The same wound isolate responded with significant differences in biofilm formation to different wound-mimicking media. Biofilm dispersal, as a proxy for effectiveness of antibiofilm treatments, was also investigated in response to proteinase K. The dispersal effect of proteinase K showed that the biofilm dispersal is contingent upon the specific wound isolate, with isolates CCUG 35571 and ATCC 6538 showing considerable dispersal responses. In conclusion, this study observed a higher biofilm formation in isolates when a protein precoating of collagen type I was applied but being dependent on the growth media selected. That is why we recommend to use simulated wound fluid or a wound-mimicking growth media to perform similar studies. Furthermore, proteinase K is suggested as an important factor that could affect biofilm dispersal within such models, since biofilm dispersal was induced in isolates CCUG 35571 and ATCC 6538 in simulated wound fluid on precoated collagen type I plates.

## Highlights

• A 2-D biofilm model developed for clinical *Staphylococcus aureus* wound isolates.
• Biofilm formation is dependent on growth medium and plate surface.
• Biofilm increases with increased protein concentration used for surface coating.
• The pre-attachment step improved biofilm formation in CCUG 44509 and ATCC 6538.
• The effect of proteinase K on biofilm dispersal is strain-specific.

## Graphical abstract

**Figure:**
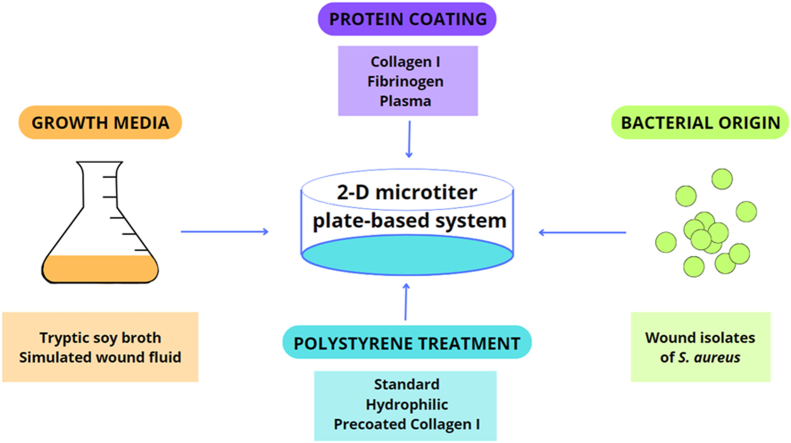

## 1 Introduction

Biofilm-associated infection (BAI) is characterised by the bacterial population being surrounded by a self-made matrix of extracellular polymeric substances (EPS) forming clumps [1]. BAI is usually related to chronic wounds, being present on them in an approximately 80 % of cases [2]. This kind of infection is particularly difficult to treat since it has an increased antimicrobial resistance and allows the pathogens to evade phagocyte attacks [3,4]. This increased resilience of biofilms comprises the intrinsic antimicrobial-resistance of each pathogen, and their combination inside a polymicrobial biofilm, offering enhanced protection to all community members. In a study by Gjødsbøl et al. [5], over 94 % of the samples collected from biofilm infections in chronic leg ulcers exhibited more than one species.

Nowadays, the standard treatment for chronic wound infections, in addition to antimicrobial therapy, is based on a mechanical debridement of the damaged tissue, which has been proven not to be completely effective against biofilm cells [6]. In order to improve the effect of some antimicrobial treatments on biofilms, in parallel with other studies aiming the inhibition of biofilm formation [7], it has been suggested to use dispersal agents in combination with antimicrobial agents. Dispersal implies the detachment of cells forming a biofilm. Dispersal agents such as DNAse, in combination with antimicrobial agents [8], and proteinase K, tested in biofilm removal [9], have previously shown to induce dispersal of biofilms. However, drug compatibility needs to be ensured, as some antimicrobials may be cleaved by proteinases.

Understanding the role of biofilms in treatment of chronic wounds is essential. For this, a realistic wound model is a need. The most common wound models are classified as *in vivo*, cell-based (or *ex vivo*) and *in vitro* models. The key advantage using *in vitro* models is the capacity to perform high throughput screening. The *in vitro* models can be of 2D type, built on a surface (usually a mutiwell plate), or 3D type, built on a scaffold. They can either contain a static medium or incorporate medium flow. All these models, as reviewed by Brackman et al. [10], have their weaknesses and strengths, with each of them being suited for a specific type of scientific questions. 2D models are usually focused on drug testing whereas 3D models are more oriented to build organoids, organ-mimicking systems. Nevertheless, it is becoming more common to use 3D models in drug testing as well [11].

The key shortcoming of the *in vitro* wound models is the poor correlation of their results with *in vivo* tests, making these models ineffective for predicting effective drug doses for human use [12]. An example of this miscorrelation is related to the discovery of Prontosil, a drug that targets a wide range of Gram-positive cocci. Even though no activity was detected on *in vitro* models, this drug was effective against pneumococcal infection *in vivo* [13]. Improving the correlation with *in vivo* tests, and thus having more reliable *in vitro* models, would help to reduce the number of required *in vivo* tests in preclinical trials, lowering the cost of assays and reducing animal suffering.

Static 2-D models are broadly used to test antimicrobial substances or evaluate biofilm formation in wound isolates. These models are popular due to their low cost and simplicity of use [12]. We chose to focus on the microtiter plate (MTP)-based static *in vitro* 2-D model, which is most commonly used to test the efficiency of antibiofilm treatments. This model can be used for a rapid quantification of formed biofilm by staining with crystal violet, safranin or congo red [14]. Other static 2-D models present some specific advantages in comparison to the MTP-based model, but they have a narrower application range [15]. Previous model testing were focused on evaluating the biofilm formation of laboratory strains [16] and evaluating the interaction between species in dual species biofilms [17].

We used the MTP-based static 2D model to evaluate biofilm formation of clinical isolates of *Staphylococcus aureus* in different growth media, with different types of protein precoating and using polystyrene-based multiwell plates with various surface properties. Proteins used for precoating were collagen type I, fibrinogen and human plasma. Collagen and fibrinogen were selected because of their abundance within the skin and connective tissue [18]. Human plasma was also chosen due to its presence within the wound environment. The effectiveness of proteinase K as a dispersal agent on biofilm dispersal was also investigated in this model [8,9].

Even though, the effect of fibrinogen or fibronectin on the biofilm formation of *S. aureus* clinical isolates was previously reported [19], in this study we used the 2D wound model to study the effect of realistic elements of the model including using clinical isolates, incorporating simulated wound fluid (SWF), as well as coating with different wound-relevant proteins (collagen, fibrinogen, and plasma) on the biofilm formation. Along with the biofilm formation of these clinical isolates, we also investigate their biofilm dispersal as the relevant step in the biofilm life cycle. The dispersal using dispersing agent proteinase K clarifies how sensitive these clinical isolates are to enzymatic breakdown of the biofilms. In fact, we first develop the 2D wound model by biofilm formation of clinical isolates, and we further disperse the biofilms using dispersal agent that can be additionally combined with antimicrobial agents treatment for wound healing in future studies.

## 2 Materials and Methods

### 2.1 Bacterial strains

Seven strains of *S. aureus* obtained from wounds were studied. CCUG 33290, CCUG 34116, CCUG 35218, CCUG 35571, and CCUG 44509 were collected from the Culture Collection of the University of Gothenburg (CCUG; Sweden) and ATCC 6538 and ATCC 29213 from the American Type Culture Collection.

The isolates CCUG 34116, CCUG 35571, and CCUG 44509 were identified by Api ID32staph identification method in the CCUG. The two missing isolates, CCUG 33290 and CCUG 35218 were not identified by the CCUG.

### 2.2 Multiwell plates made of various surface properties and coated with various proteins

Four different polystyrene-based microwell plates with different surface properties were used during this study: standard polystyrene and hydrophilic polystyrene (Thermo Fisher Scientific), hydrophilic polystyrene (TPP), and precoated with collagen type I (rat tail, gibco). All the plates were in a 96-well format.

Three different proteins were used for plate precoating: collagen type I (rat tail; >90 %; gibco), fibrinogen (human; >90 %; Sigma Aldrich), and plasma (human; checked for hepatitis B + C and VIH; Sigma Aldrich). Collagen type I was diluted in acetic acid (HAC; 30 %; Sigma Aldrich) 0.25 % whereas fibrinogen and human plasma were diluted in phosphate-buffered saline (PBS). 200 μL were added in each well and incubated overnight at 8 °C, according to the manufacturer's instructions (Collagen Attachment Protocols, Solubility, and Stability https://www.sigmaaldrich.com/SE/en/technical-documents/technical-article/cell-culture-and-cell-culture-analysis/mammalian-cell-culture/collagen-product-protocols). Thereafter, each well was rinsed 3 times with 200 μL of PBS.

### 2.3 Static wound model

The bacterial samples were grown in 3 mL of tryptic soy broth (TSB) incubated at 35 °C overnight. Then, the bacterial culture was diluted to OD_565_ ∼0,9 (DEN-1B grant-bio) in Fys NaCl + Peptone 0.1 % (PW) and added to the media (1:100 dilution), resulting in 10^6^ colony-forming units (CFU)/mL approximately. Two different media were used: simulated wound fluid (SWF; Sahlgrenska substratavdelning), based on 50 % PW and 50 % foetal bovine serum, and TSB. The intention of using SWF was to reflect the protein content in chronic wound fluid [20].

150 μL of the initial inoculum were added in each well and incubated for 24 h at 35 °C with 90 % relative humidity. The humidity was controlled with a humidity sensor inside the incubator, to prevent the wound model from drying. In addition, it has been shown that moist conditions are favourable in wound healing [21].

After the first 3 h of the 24-h incubation the pre-attachment step was performed. The bacterial solution was removed from the wells by pipetting out the liquid. The non-attached bacteria were rinsed twice with PBS. 150 μL of SWF were added in each well to continue the incubation of bacteria attached in the wells for another 21 h.

### 2.4 Quantification of biofilm formation and cell counting during biofilm dispersal

The quantification of biofilm formation was done using crystal violet (CV). After 24-h incubation at 35 °C with 90 % relative humidity, the bacterial solution was removed from the wells by pipetting and 175 μL of CV 0.05 % (aqueous, Sigma Aldrich) were added on the top of formed biofilms for 5 min at room temperature, modifying the protocol used by Zulfakar et al. [22]. Then, the stain was removed by pipetting and plates were dipped in water twice and dried under laminar air flow. 200 μL of acetic acid (HAC) 30 % were added over the dried samples to solubilize the staining and measure its absorbance on PowerWave HT (BioTek) at 590 nm. The OD at 590 nm was measured in wells with only growth medium. The mean value of these measurements was subtracted from all the measurements as the background level.

In addition, following dispersal experiment using proteinase K, the CFU counting was used to estimate the number of cells. 3 M AC Petrifilm was used to do the counting in 3 M Petrifilm Plate Manager. Dispersed cells were collected by pipetting from the wells and diluted in PW. 1 mL of this solution was used for each petrifilm which was incubated at 35 °C overnight before counting. Counting dispersed cells from wells without proteinase K were used as negative control.

### 2.5 Proteinase K

Proteinase K as the dispersal agent was diluted in SWF at different concentrations, from 10 μg/mL to 100 μg/mL. The concentrations were selected based on a previous study of proteinase K effect on biofilm removal [9]. After 24-h incubation at 35 °C with 90 % relative humidity, biofilms were rinsed twice with PBS and 150 μL of the proteinase K solution were added in each well and newly incubated at 35 °C with 90 % relative humidity for 2 h.

To reduce the number of experimental variables, the precoated collagen type I plates were chosen for this experiment. OD_590_ measurement was used to measure the biofilm dispersal and CFU counting was used to distinguish dispersal from cell death.

### 2.6 Statistical analysis

All data were plotted using mean ± standard deviation (SD). The comparison of biofilm formation on different isolates was carried out by Tukey's multicomparison test. The analyses were performed in GraphPad Prism 10 using two-way analysis of variance (ANOVA) and XY correlation studies. An alpha parameter of 0.05 was used.

## 3 Results and discussion

### 3.1 Protein-coating improved the biofilm formation of wound isolates

**Fig. 1:**
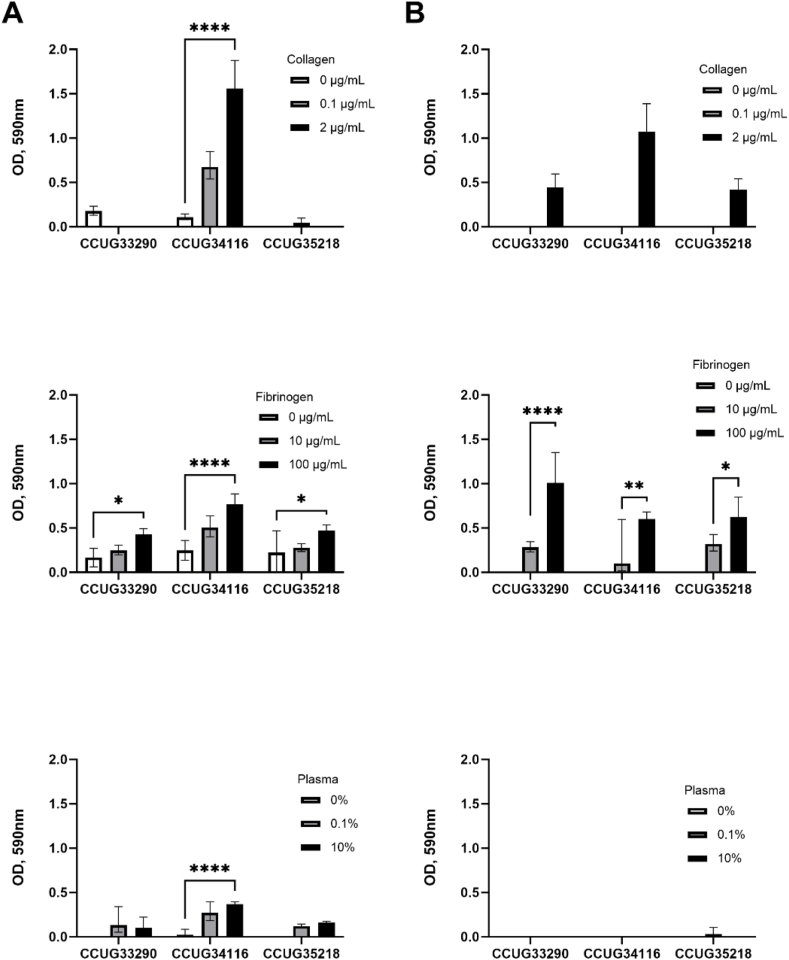
Evaluation of biofilm formation measured as optical density (OD) at 590 nm of isolates CCUG 33290, CCUG 34116, and CCUG 35218 of *S. aureus* grown on surfaces coated with different proteins. The results were compared with control (0 μg/mL or 0 % of relevant protein). A) Biofilm formation measured as OD at 590 nm in TSB medium versus B) biofilm formation in SWF medium. ** = p < 0.01, *** = p < 0.001, **** = p < 0.0001. n = 3.

**Fig. 2:**
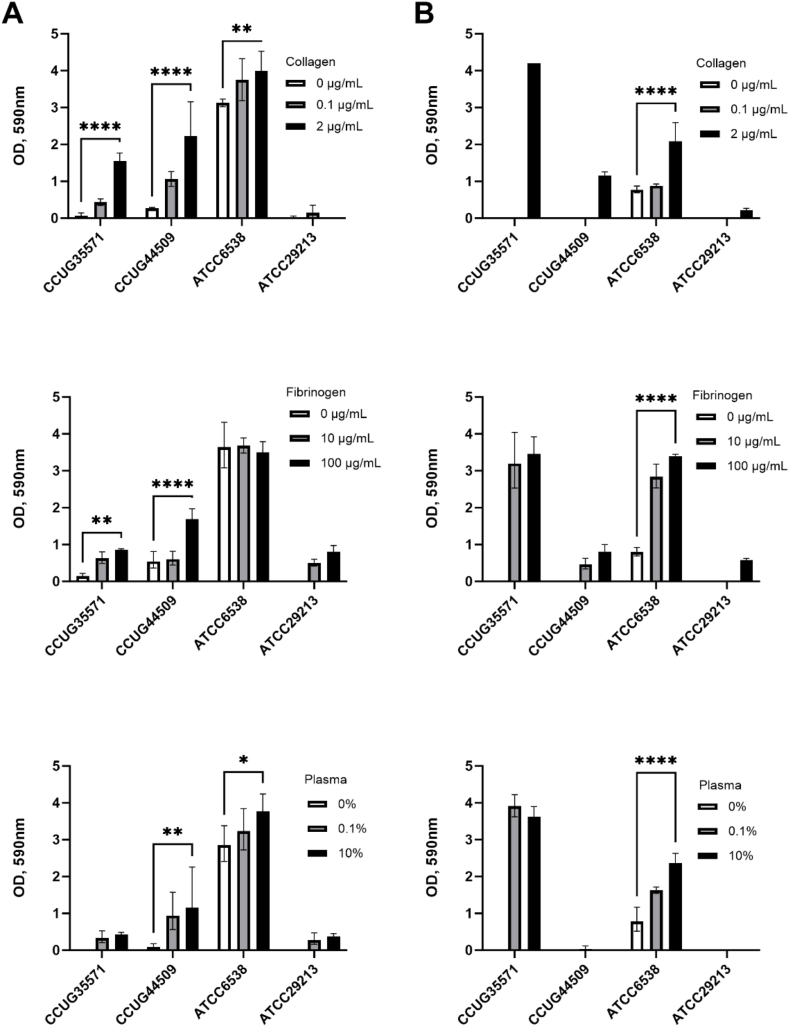
Evaluation of biofilm formation measured as optical density (OD) at 590 nm of isolates CCUG 35571, CCUG 44509, ATCC 6538, and ATCC 29213 of *S. aureus* grown on surfaces coated with different proteins. The results were compared with control (0 μg/mL or 0 % of relevant protein). A) Biofilm formation measured as OD at 590 nm in TSB medium versus B) biofilm formation in SWF medium. ** = p < 0.01, *** = p < 0.001, **** = p < 0.0001, n = 3.

To study the enhancement of bacterial adhesion by protein precoating, we used collagen type I (at 0, 0.1 and 2 μg/mL), fibrinogen (at 0, 10 and 100 μg/mL) and human plasma (at 0, 0.1 and 10 %). The protein concentrations were selected based on a previous optimization in our laboratory (data not shown). Bacterial isolates were grown in two different media using TSB (Fig. 1A and Fig. 2A) and SWF (Figs. 1B and 2B) to check the effect of growth medium on the biofilm formation. Wells with non-contaminated medium were used as the background. The results were compared with control (0 μg/mL or 0 % of relevant protein).

CV staining of the biofilms obtained from isolates CCUG 34116, CCUG 35571, CCUG 44509 and ATCC 6538 showed a significant change in biofilm formation in relation to protein concentration. The highest biofilm formation was exhibited by strains CCUG 35571 on SWF and ATCC 6538 on both media. For most strains, an increase in protein correlated to increased biofilm formation. In addition to this, taking as an example strain CCUG 35571, it was observed that the growth medium affects the biofilm formation capability. For this strain, biofilm formation varies four-fold between two media, with OD_590_ of 1 in TSB, and OD_590_ of 4 in SWF (Fig. 2).

As collagen is a largely hydrophobic molecule, we tested the effect of surface precoating with collagen over different types of well plates including standard polystyrene and hydrophilic polystyrene (Thermo Fisher Scientific), hydrophilic polystyrene (TPP). In all tested plates, the effect of surface coating with collagen was similar among all tested plates including standard and hydrophilic ones. It means that all tested clinical isolates (CCUG 34116, CCUG 35571, CCUG 44509 and ATCC 6538) had stronger biofilm formation capacity over collagen than the non-coated plates (Fig. S1).

These results suggest that wound models should prioritize wound-mimicking media to produce more accurate results. The observed influence of protein precoating on increased biofilm formation by most strains suggests that a reliable wound model should mimic the protein network present in the wound. This network is arguably crucial for bacterial attachment and, consequently, colonization.

Collagen has been independently validated in static wound models, mainly in matrix gel form. A study made by Werthén et al. [23] reported similarities in the biofilm structures formed in this model and those formed *in vivo*. Our results support this conclusion and suggest collagen is a valuable base for MTP-based static *in vitro* 2D model.

### 3.2 Impact of multiwell plates made of various surface properties on biofilm formation

Based on the results presented in (Figs. 1 and 2), CCUG 34116, CCUG 35571, CCUG 44509, and ATCC 6538 were selected for further experiments based on their high biofilm formation capacity in different media. SWF was selected as the preferred medium, due to its higher clinical relevance in wounds compared to TSB, and better growth of clinical isolates (Figs. 1 and 2). Due to this media selection, the isolate CCUG 44509 was discarded due to its low biofilm formation in SWF (Fig. 2B). Collagen type I was selected as the preferred coating. The biofilm formation capacity of the selected isolates was examined in polystyrene-based multiwell plates with various surface properties. Standard polystyrene, hydrophilic polystyrene and TPP hydrophilic polystyrene plates were selected and coated with collagen type I (2 μg/mL). Plates precoated with collagen type I were used as the positive control and wells with non-contaminated medium were used as a negative control (background). This background level was subtracted from all measurements.

**Fig. 3:**
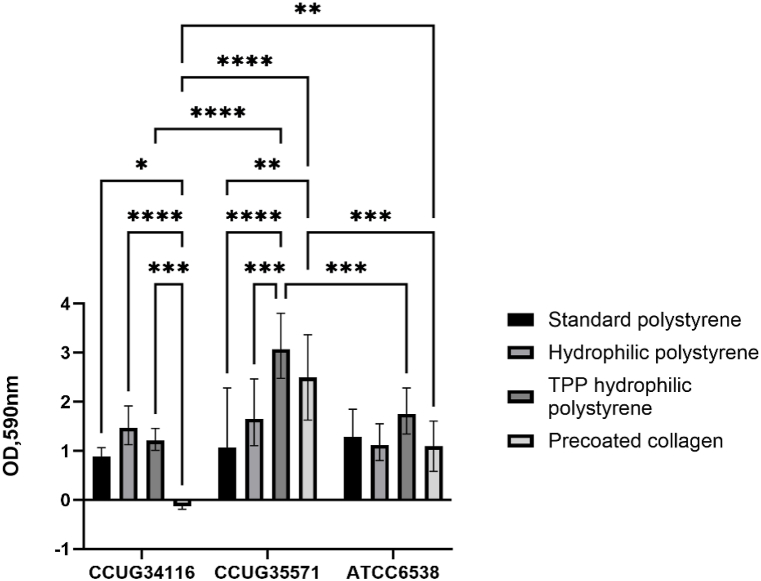
Evaluation of biofilm formation measured as optical density (OD) at 590 nm of selected strains of *S. aureus* grown in SWF medium and on polystyrene-based multiwell plates with various surface properties coated with collagen. The background level, measurements in negative controls, was subtracted in all measurements. * = p < 0.1, ** = p < 0.01, *** = p < 0.001, **** = p < 0.0001, n = 5.

The isolate CCUG 35571 was the most sensitive to the surface material of tested plates (Fig. 3). The isolate CCUG 34116 showed less pronounces but significant differences. Contrarily, the isolate ATCC 6538 showed no significant differences with different surfaces.

The biofilm formation on TPP hydrophilic polystyrene increased significantly in isolate CCUG 35571 compared to CCUG 34116 and ATCC 6538. Moreover, the results on precoated collagen also showed quantitative differences between isolates, reaching OD_590_ of 2,5 in case of CCUG 35571 but not surpassing the background level within CCUG 34116. Based on these results, biofilm formation in relation to multiwell plate surface with various properties is extremely strain-specific, and this can be a very important factor in choosing the correct experimental setup for 2D assays.

### 3.3 The pre-attachment step improved biofilm formation for strains CCUG 44509 and ATCC 6538

**Fig. 4:**
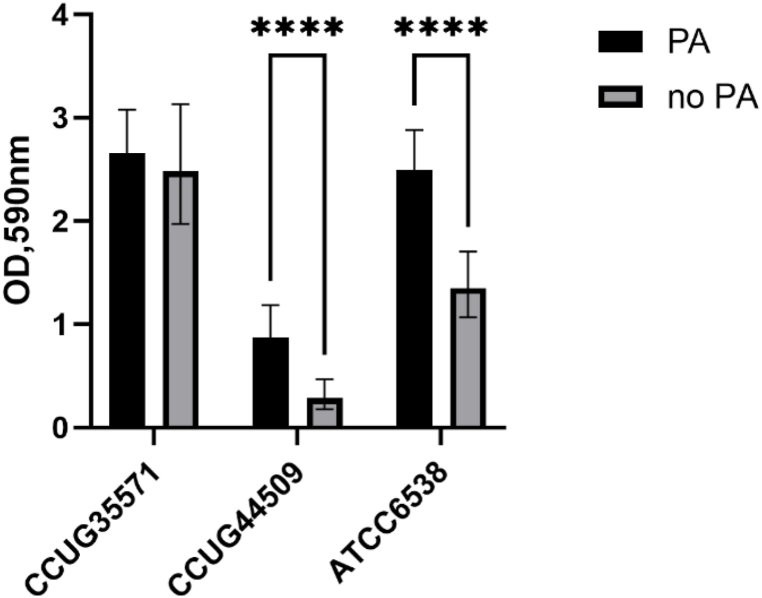
Evaluation of the effect of PA on biofilm formation measured as optical density (OD) at 590 nm of selected strains of *S. aureus* grown in SWF medium on precoated collagen type I plates. The biofilms were stained with crystal violet followed by absorbance measurement. **** = p < 0.0001, n = 42.

The same three isolates used in the plates comparison were examined to determine the effectiveness of pre-attachment step (PA) on biofilm formation (Fig. 4). The isolate CCUG 44509 was included again, despite of having low biofilm formation in SWF (Fig. 2B), to test the pre-attachment step efficacy. In contrast, the isolate CCUG 34116 was discarded due to its low biofilm formation over precoated collagen plates (Fig. 3).

This PA step, as described in Materials and Methods, was added to form the biofilm only with the bacteria which were already attached to the surface within the first 3 h of incubation. Based on the study by Li et al. [24], there are two kinds of attachment between the surface and bacteria: reversible and irreversible. The irreversible attachment happens after reversible attachment, and it marks the point when cell-surface interactions prevail and, consequently, biofilm formation start. We wanted to assess if PA can increase the biofilm formation capacity of isolates. All strains were grown on precoated collagen type I plates and wells with non-contaminated medium as the background. SWF was the growth medium used in this experiment.

In this assay, biofilm formation of CCUG 35571 was not affected by PA. By contrast, ATCC 6538, which is also a strong biofilm former, was positively affected by PA (Fig. 4). Similar behaviour was seen in CCUG 44509. This weak biofilm former increased its biofilm formation by PA.

### 3.4 Proteinase K reduced biofilm formation

Based on the abovementioned results, we further examined the strongest biofilm formers, CCUG 35571 and ATCC 6538, for sensitivity to the dispersal agent proteinase K. Since biofilm dispersal is relevant within the biofilm life cycle, this study on proteinase K wanted to observe the effect of the culture conditions and strain-selection on how resistant the biofilms were against enzymatic breakdown. To induce dispersal and force bacteria to convert from biofilm to planktonic mode, we investigated the effect of different concentrations of proteinase K. The results were compared with control (0 μg/mL proteinase K).

**Fig. 5:**
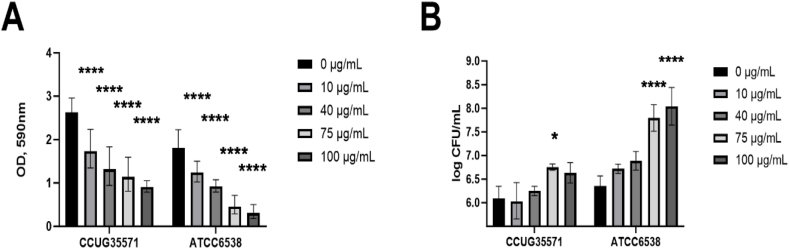
Evaluation of the effect of different concentrations of proteinase K diluted in SWF over the biofilms for 2 different strains for 2 h in the incubator. The results were compared with control (0 μg/mL proteinase K). A) Optical density (OD) at 590 nm and B) CFU from this experiment. * = p < 0.1, **** = p < 0.0001, n = 9 (A) and 3 (B).

Proteinase K at all concentrations induced a significant decrease in biofilm formation of both isolates CCUG 35571 and ATCC 6538, which was concentration dependent (Fig. 5A). This suggested that proteinase K successfully induced biofilm dispersal. Next, biofilm dispersal was further confirmed by CFU counts in the supernatant (Fig. 5B). The number of viable bacterial cells in the supernatant was directly proportional to the increment of proteinase K concentration (Fig. 5B).

Our result on proteinase K inducing biofilm dispersal was in agreement with previous results [9]. An additional insight came from using clinical isolates in our study, which gave slightly different results in the dispersion assay. While the strain ATCC 6538 exhibited perfect alignment between biofilm reduction and CFU counts, this correlation was not so obvious for the isolate CCUG 35571. Cells of CCUG 35571 were more susceptible to proteinase K, leading to reduced CFU counts in the supernatant. This observation implies that when applying an antimicrobial treatment together with a dispersal agent, the optimal quantity of the dispersal agent and the duration of treatment may need to be adapted for eradicating different strains of the same pathogen species.

This finding emphasizes the need for studying antimicrobial treatments with dispersal agents in suitable wound models, where duration of interaction and concentration of the dispersal agent should be optimized.

## 4 Conclusions

In this study, we first examined the impact of realistic elements of 2D wound model on biofilm formation. We used wound clinical isolates, incorporated SWF to the model, and performed surface coating with wound related proteins (collagen, fibrinogen, and plasma). Our results showed that some of the isolates (CCUG 34116, CCUG 35571, CCUG 44509, and ATCC 6538) were sensitive to the variations in protein concentration used for surface coating. Collagen and fibrinogen were also found as the valuable bases for MTP-based static *in vitro* 2D model. The positive effect of the pre-attachment step on biofilm formation was shown in some isolates (ATCC 6538 and CCUG 44509), but it was not universally observed. In case of the biofilm dispersal as the relevant step in the biofilm life cycle, both tested isolates (CCUG 35571 and ATCC 6538) biofilms were dispersed in response to proteinase K. However, the CCUG 35571 biofilm dispersal did not yield as many cells as ATCC 6538 biofilm, that clarifies how sensitive ATCC 6538 isolate is to enzymatic breakdown of the biofilms. The biofilm dispersal sensitivity of isolate can be combined with antimicrobial therapy to improve the wound healing process. The proposed simple and cost-effective 2D wound model can also be applied for initial screening of antimicrobial substances against *S. aureus* clinical isolates.

## Data availability

No data was used for the research described in the article.

## CRediT authorship contribution statement

**José Manuel Outomuro Ruiz:** Writing – original draft, Visualization, Validation, Formal analysis. **Erik Gerner:** Writing – review & editing, Supervision, Methodology, Investigation. **Shadi Rahimi:** Writing – review & editing, Supervision, Project administration. **Leonarda Achá Alarcón:** Writing – review & editing, Supervision. **Ivan Mijakovic:** Writing – review & editing, Supervision, Funding acquisition, Conceptualization.

## Declaration of competing interest

José Manuel Outomuro Ruiz and Erik Gerner were employed by Mölnlycke Health Care AB by the time this study was done, as a master student and employee respectively.
