# Supplementary material for: Biofilm formation and dispersal of Staphylococcus aureus wound isolates in microtiter plate-based 2-D wound model
Source: Heliyon. 2024 Jul 2;10(13):e33872. doi: 10.1016/j.heliyon.2024.e33872 (PMC11269870; doi:10.1016/j.heliyon.2024.e33872)
Supplement: Multimedia component 1 [file mmc1.docx]

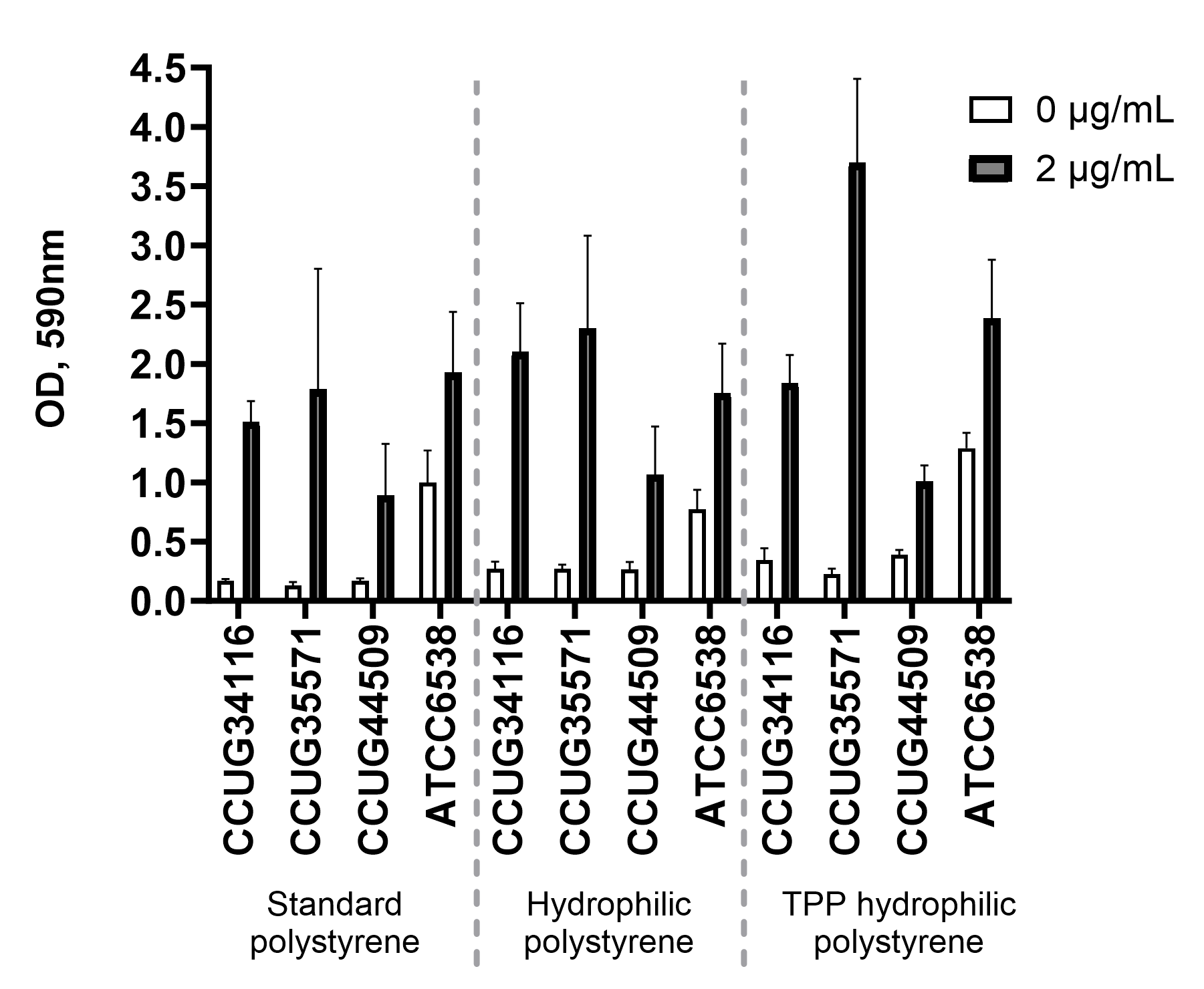


Figure S1: Evaluation of the effect of protein precoating with collagen type I over standard polystyrene and hydrophilic polystyrene (Thermo Fisher Scientific), hydrophilic polystyrene (TPP)-based multiwell plates using wound isolates of S. aureus. The results were compared with control (0 µg/mL collagen type I). n = 5.
